# Impact of Sustainable Manufacturing Processes on the Rheological and Microstructural Stability of Biopolymer-Stabilized Oil-in-Water Emulsions

**DOI:** 10.3390/gels12030211

**Published:** 2026-03-04

**Authors:** Marlène Lartigue, Claire Dang, Céline Saure, Sophie Cambos, Alicia Roso

**Affiliations:** 1 Seppic European Customer Technical Service Center, 127 Chemin de la Poudrerie, 81100 Castres, France; marlene.lartigue@airiquide.com (M.L.);; 2 Seppic Research and Innovation, 127 Chemin de la Poudrerie, 81100 Castres, France

**Keywords:** bio-based polymers, emulgel, energy-efficient production one-pot, hot-cold, flow behavior, gel structure, stability

## Abstract

This work investigated the impact of energy-efficient and water-saving manufacturing procedures—specifically one-pot and hot-cold processes—on the rheological and microstructural stability of oil-in-water (O/W) emulsions (emulgels) stabilized by four distinct biopolymers and benchmarked against a synthetic polymer. Emulgels produced using these sustainable methods were directly compared against a traditional hot process. Results demonstrated that for most biopolymers, including tara gum, glucomannan, and cross-linked xanthan gum, the sustainable manufacturing procedures did not compromise overall stability and often provided beneficial polymer-specific flow profiles, such as reduced thixotropy or enhanced shear-thinning. A notable exception was the co-processed acacia/xanthan gum, where rheological data indicated that the one-pot process should be avoided due to structural degradation. Collectively, these findings broaden the applicability of sustainable manufacturing methods beyond traditional stabilizers like xanthan gum and provide additional data for process optimization, with tentative suggestions for transferability to food emulgel production.

## 1. Introduction

Polymeric rheology modifiers or thickeners are essential for the stability and sensory profiles of topical oil-in-water (O/W) emulsions [1], the most prevalent vehicle in skincare [2]. When stabilized by polymer networks, these systems—often referred to as emulgels, or emulsion gels, in pharmaceutical and food literature—exhibit defined viscoelastic properties [3]. Emulgels are prepared using various gelling agents—such as synthetic (known to provide high viscosity at low concentrations), semi-synthetic (e.g., cellulose derivatives often used for their film-forming properties), and biopolymers (e.g., xanthan gum, alginates or pectin, selected for their biocompatibility)—to improve the stability of active ingredients and modulate their skin release or to texturize foods [4,5,6]. Flexibility of polymer introduction is an important asset in production, as a single ingredient can be used in multiple types of formulations and purposes for economic and sustainability considerations. While synthetic polymers, such as acrylic and acrylamide-based rheology modifiers, offer manufacturing flexibility [7,8], consumer demand and environmental goals are driving a shift toward bio-based alternatives. The cosmetic industry is increasingly utilizing natural polymers, especially polysaccharides, which were extensively studied in recent years as alternatives to synthetic polymers for stabilization, thickening, film formation, and sensory properties [9,10,11,12].

The transition to natural polymers must align with broader sustainability goals, particularly the reduction of energy and water consumption during manufacturing. Traditional oil-in-water (O/W) emulsion processes require significant thermal energy, with heating and cooling operations accounting for over 90% of the total energy footprint [13,14]. Consequently, energy-efficient methodologies such as the hot-cold process (which reduces thermal energy by limiting the heated volume) have gained prominence. The production with sequential ingredient addition in one vessel, called the one-pot process, which minimizes water waste by eliminating sequential vessel cleaning, is a meaningful alternative, particularly when large-scale production volumes are targeted, such as body care or hair care formulations. However, the importance of investigating these processes lies in their potential impact on polymer behavior. Biopolymers typically suffer from poor dispersibility, traditionally requiring either high-shear pre-dispersion in separate vessels or prior preparation as a slurry in a glycol [15]. Bypassing these steps via sustainable processes introduces new challenges, such as competitive migration at the oil-water interface or insufficient polymer swelling, which can compromise the final microstructural stability of the emulgel. Previous investigations, using the hot-pot process, highlighted the potential of compositions containing less than 50% of oil [16] and confirmed the process performance with a natural composition combining glyceryl esters with xanthan gum to emulsify 20% of a sunflower seed oil [14]. The suitability of the one-pot process for the production of natural O/W emulsions was recently reported. The formulations included a waxy emulsifier (3 to 8%), xanthan gum at 0.3%, and two types of emollients from 10 to 30% (Caprylic/capric triglyceride or octyldodecanol). A more complex formulation was also prepared with tara gum at 0.5% [17]. Overall, the applicability of these two process options to reduce environmental impacts using biopolymers as thickeners/stabilizers remains scarce, except for xanthan gum.

Therefore, this work aimed to investigate the impact of energy-efficient manufacturing procedures of an O/W emulsion using biopolymers from different resources and with various properties (thickening and/or stabilizing, film-forming or not), compared to a synthetic polymer reference. To understand how sustainable processing impacts critical networks of emulgels, four hydrocolloids, representing distinct structural architectures, were selected to represent different strategies for achieving viscosity and stabilization in aqueous dispersions. Tara gum (galactomannan polysaccharides) [18] and a glucomannan extracted from the roots of *Amorphophallus muelleri* represent systems driven by physical entanglement, providing a significant thickening effect in their native states. An experimentally modified cross-linked xanthan gum was selected to represent chemical networking, forming a covalent hydrogel that improves sensory characteristics [19]. Finally, a co-processed blend of *Acacia senegal* gum and xanthan gum exemplifies functional blending. In this system, xanthan gum provides the bulk rheological network, while Acacia senegal acts at the interface to stabilize potential oil or hydrophobic components [20,21]. In water, it forms a “structured fluid” with film-forming properties [22], distinct from the gelling agent or thickeners above. By examining parameters such as macroscopic stability, flowing profiles, and viscoelastic properties, this work aims to provide data for process optimization and assess the versatility of these sustainable manufacturing methods.

## 2. Results and Discussion

Emulsion compositions were selected to be representative of minimalist skincare emulsions (i.e., few ingredients, reduced dosage of oil phase and surfactant). The selected waxy glucolipid surfactant (requiring heating to 80 °C for complete melting) represents a worst-case scenario for sustainable emulsion manufacture, as both the hot-cold and one-pot processes pose additional challenges in achieving complete melting. This emulsifier was known to promote lamellar phases [23] with a combination of both liquid-crystalline and α-crystalline gel phases. However, the emulsifier concentration was intentionally selected to provide insufficient formulation macroscopic stability, to better investigate the influence of the formulation manufacturing procedure on the stabilizing effects of the tested polymers. The polymers were investigated at their respective average recommended concentrations to achieve optimum stability of the formulations (Composition available in Table 1 in the next section). The intention is to focus on the influence of the manufacturing process rather than comparing the emulsion characteristics across the biopolymers, as they may be used for different purposes.

Three distinct manufacturing processes were investigated for each polymer in the laboratory. A direct addition of the polymer into the heated oil phase in the main tank was chosen as the basic procedure (termed ‘indirect’). This approach, which was not optimized for energy consumption, was selected because it offers progressive hydration by separating the polymer particles, allows for versatile composition without glycols or other powders, and simplifies the process by avoiding prior preparation in an additional vessel. Two processes aimed at reducing environmental impacts (carbon footprint and water consumption) were attempted: one-pot and hot-cold, with one-third of the water added cold (detailed manufacturing processes are described in Section 4.2).

### 2.1. Impact of Processing Conditions on Emulsion Characteristics and Physical Stability

**Figure 1:**
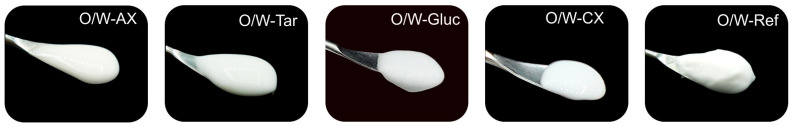
Emulsions’ visual texture and exemplification with indirect manufacturing procedure after 7 days of storage at room temperature (20 °C ± 2 °C).

Variations in the manufacturing process did not affect the emulsion’s visual appearance, qualitative observations of flowability, or the pH of the formulations (Summary in Table 1). The texture (qualified by visual observation and illustrated in Figure 1) and Brookfield Viscosity of the emulsions (Table 1) depended on the selected polymer, as expected from their nature and varying concentrations, but no significant differences were observed between the manufacturing processes at Day 7 after manufacturing. The bulk texture of the emulsions was perfectly smooth, without any lumps, regardless of the manufacturing process and polymer nature. Emulsions exhibiting the most consistent texture were produced with glucomannan (O/W-Glu) and the synthetic reference (O/W-Ref), correlating with their respective Brookfield Viscosity values. All emulsions were physically stable after one month of storage at room temperature and 45 °C, with the exception of a significant Brookfield Viscosity increase observed in the tara gum (O/W-Tar) one-pot process after one month of storage, reaching 24,000 mPa·s. This 26% increase was not considered a cause for rejection regarding formulation stability, aligning with prior work reporting a 24% increase in similar natural O/W emulsions using a standard emulsification procedure [17].

**Figure 2:**
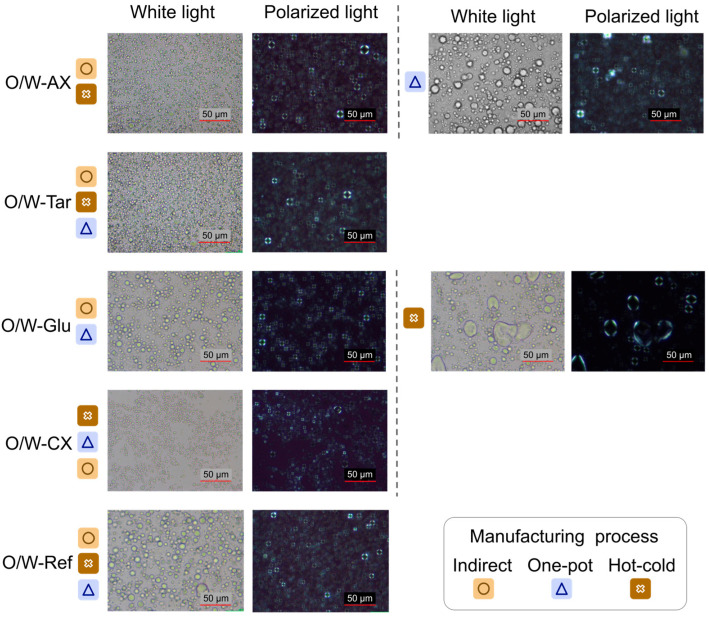
Microscopic appearance of emulsions depending on polymer and manufacturing procedure (white light and polarized light at 40× magnification; scale bar: 50 µm; 7 days after manufacturing). For visual simplification, similar observations were grouped and illustrated with one representative set of pictures. All analyses were performed at 20 °C ± 2 °C.

The manufacturing process did not impact the formation of liquid crystals (bilayers around the oil droplets) visualized as maltese cross microstructures under polarized light (Figure 2). Emulsions produced with tara gum (O/W-Tar), the experimental cross-linked xanthan gum (O/W-CX), and with the synthetic control (O/W-Ref) showed similar microscopic appearance across the different manufacturing processes. In contrast, the earlier work with tara gum [17] resulted in coarser one-pot emulsions. These varying results may be linked to the difference in the nature of the surfactants. For the two other biopolymers, particle size was more or less fine, depending on the process and the polymer’s nature. The emulsions produced with the co-processed acacia gum/xanthan gum (O/W-AX) provided a lower particle size with the two processes in which the polymer was introduced in the oil (indirect and hot-cold). This observation was consistent with the separation of polymer particles and their better dispersion in water, allowing for both a more progressive creation of the gel network and a better dispersion of the oil droplets in the emulsion. A second hypothesis suggests that the larger particle size resulting from the one-pot process was specifically linked to the polymer’s nature and the selected high dosage. The rapid dissolution of the highly branched, water-soluble acacia gum, which coats the xanthan gum, results in very rapid swelling (in less than 5 min, compared to over 30 min for isolated xanthan gum; Table A1, Appendix A), which could create a competitive effect. In the one-pot process, the simultaneous presence of oil and melted surfactant, coupled with the acacia gum’s intrinsic surface-active properties, would likely cause the acacia component to be prematurely adsorbed at the oil-water interface. This competitive migration could then inhibit the inner core xanthan gum from fully swelling, consequently hindering the formation of a robust gel network and resulting in micro-agglomerates undetectable by visual inspection. However, the use of a Brookfield viscometer provided insufficient detail to detect the viscosity differences necessary to support this hypothesis, which is further analyzed in light of rheology results. For glucomannan, the emulsions (O/W-Glu) imparted a lower particle size with indirect and one-pot processes. For O/W emulsions, an increased Brookfield Viscosity by adding polymers during high shear emulsification with a conventional hot process generally results in increased shearing and improved oil droplet dispersion [24,25]. However, in the case of the hot-cold process, the initial use of only two-thirds of the water could create an overly high polymer concentration and excessive consistency. This high viscosity would then hinder the surfactant’s migration to the interfaces, which could result in a coarser emulsion. This hypothesis was supported by the specific high Brookfield Viscosity of the glucomannan in water at the corresponding concentration (Table A1 in Appendix A).

### 2.2. Impact of Processing on Emulsion Flow Properties

**Figure 3:**
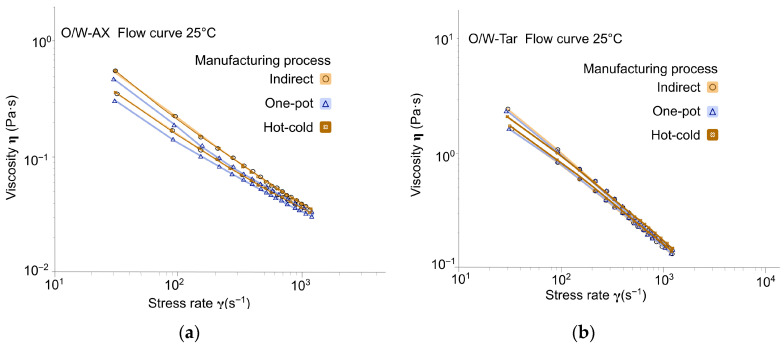
Flow curves across manufacturing processes (Day 7, at 25 °C ± 2 °C): (**a**) Emulgels with co-processed acacia gum/xanthan gum: O/W-AX; (**b**) Emulgels with tara gum: O/W-Tar.

**Figure 4:**
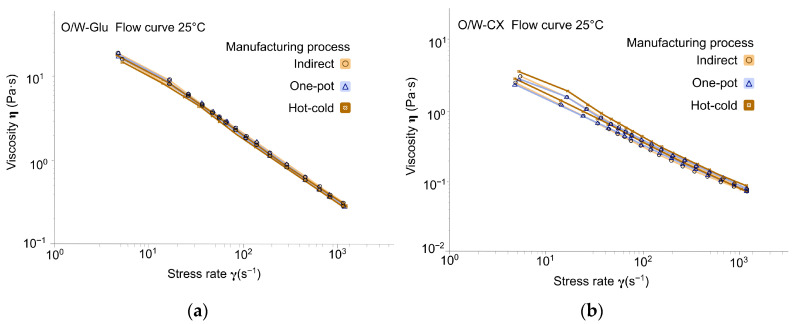
Flow curves across manufacturing processes (Day 7, at 25 °C ± 2 °C): (**a**) Emulsions with glucomannan: O/W-Glu; (**b**) Emulgels with cross-linked xanthan gum: O/W-CX.

**Figure 5:**
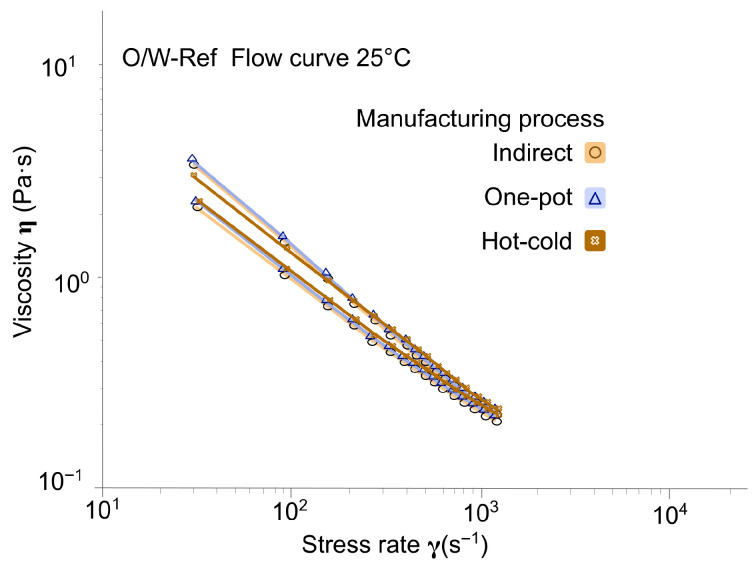
Flow curves of synthetic polymer emulgels across manufacturing processes (O/W-Ref), (Day 7, at 25 °C ± 2 °C).

The decreasing viscosity as a function of shear rate, evident from the slope of the flow curves (Figure 3, Figure 4 and Figure 5), demonstrated that all biopolymer-stabilized emulsions exhibited shear-thinning behavior. As expected from the different compositions and polymer concentrations, the shear-thinning behavior, the presence or absence of a yield stress, and the magnitude of the hysteresis loop varied depending on the chosen polymer, regardless of the manufacturing process. Aqueous dispersion of the polymers at the same or a close corresponding concentration also exhibited shear-thinning character, but differed from the emulsions in their lack of visible thixotropy (Table A2 in Appendix A).

Based on similar yield stress (around 3 Pa) and Flow Behavior Index values (around 0.6) in Table 2, the one-pot and hot-cold processes did not affect the shear-thinning profile of the co-processed acacia gum/xanthan gum emulgel (O/W-AX; Table A2; Figure 3a) compared to the indirect process. The results indicated that the co-processed acacia gum/xanthan gum formed a continuous phase gel network that traps the oil droplets, thereby supporting formulation stability. However, the one-pot process showed a significantly lower hysteresis loop area. This area represents the required energy dissipated per unit volume to break down the structure established by that specific process. Since the chemical composition is identical, this difference can suggest the formation of a weaker gel network density, which is easier to break down. As the viscosity was also significantly lower (0.5 Pa·s at 16.3 s^−1^ instead of ≌0.7 Pa·s for the other two procedures), contrary to what was previously observed with the Brookfield viscometer device in the previous section (Figure 2), it can be hypothesized that while the stabilization mechanism did not change, the network strength was reduced. Combined with the suboptimal particle dispersion observed in the microscopic examination (Figure 2), it is more probable that the too-fast dissolution of acacia gum prevented the associated xanthan gum from fully swelling in the presence of a blend of oil and emulsifier.

For Tara Gum (O/W-Tar), similar emulgel flow characteristics were obtained between the one-pot and indirect processes, with a yield stress of approximately 3, a pronounced shear-thinning character and a noticeable thixotropy (Table 2; Figure 3b). When handled with the hot-cold process, the only difference was a significant decrease in the hysteresis loop area, without a decrease in viscosity.

Glucomannan resulted in an identical profile across the three manufacturing processes, as demonstrated by the superposition of the curves (Figure 4a) and the similarity of the corresponding indicators in Table 2. Emulsion behavior is close to the boundary between liquid and gel structure (emulgel), characterized by an absence of visible yield stress (consistent with the corresponding aqueous gel; Table A3 in Appendix A), and a low, non-thixotropic, shear-thinning behavior (Flow behavior index ≌ 0.8). These flow properties were consistent with the viscoelastic data presented in Section 2.3.

For cross-linked Xanthan (O/W-CX), variations of the manufacturing process led to several significant differences (Figure 4b): a decrease in yield stress and a greater degree of shear-thinning (Flow behavior index dropped from 0.68 to 0.45–0.47, Table 2), associated with a decreased hysteresis loop area characterized the emulgel obtained with the one-pot procedure. Compared to the corresponding aqueous gel (hydrogel) (Table A3 in Appendix A), the emulsions exhibited a significant increase in yield stress, regardless of the manufacturing process (from 1.40 Pa in the gel to 11.78 to 23.03 Pa in emulsions), which was not the case for the other polymers. These findings suggested higher interactions with the surfactant, resulting in a more organized gel network [16].

Despite not being the core subject of the investigation, emulgels stabilized with the synthetic reference (O/W-Ref, Figure 5) also showed significant differences in behavior under stress depending on the manufacturing process, especially in a less-thick emulsion with the one-pot and hot-cold processes. The drop in hysteresis loop area was hypothesized to be linked to this significant decrease in apparent viscosity, as shown by values at 16 s^−1^ decreasing from 5.69 Pa·s (indirect) to 4.70 Pa·s and 4.54 Pa·s (one-pot and hot-cold, respectively). Similar behavior was reported in a previous study [14] involving acrylate copolymer stabilizer: the standard hot process yielded a higher low-shear-rate viscosity than the hot-cold process. This outcome was attributed to the shorter cooling time of the hot-cold method, which reduced the tendency for surfactant gel-phase organization.

Flow rheological profiles provide insights into emulsion behavior under stress, such as those encountered during packaging filling or application to the skin [26,27]. For tara gum emulgels, the hot-cold process yielded lower hysteresis with maintained viscosity, facilitating faster structural recovery for industrial filling lines. For cross-linked xanthan gum emulgels, the one-pot and hot-cold processes resulted in lower yield stress and higher shear-thinning, which could be advantageous for pouring from narrow-aperture packaging (e.g., make-up removal lotion). For the remaining biopolymers, the slight process-dependent differences observed suggest a minimal impact on stressing operations during use, such as spreading on the skin (corresponding shear range: 500 to 10,000 s^−1^ [28]).

### 2.3. Impact of Processing on Emulgel Viscoelastic Properties

**Figure 6:**
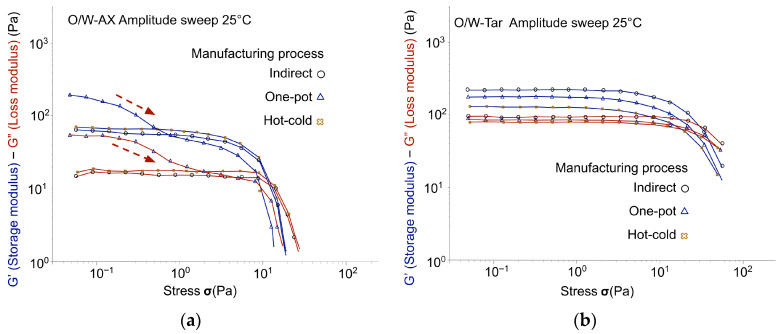
Oscillatory stress sweep: G′ and G″ moduli across manufacturing processes (Day 7, at 25 °C ± 2 °C): (**a**) Emulgels with co-processed acacia gum/xanthan gum: O/W-AX with highlighted impact of one-pot process on the linear viscoelastic region; (**b**) Emulgels with tara gum: O/W-Tar.

**Figure 7:**
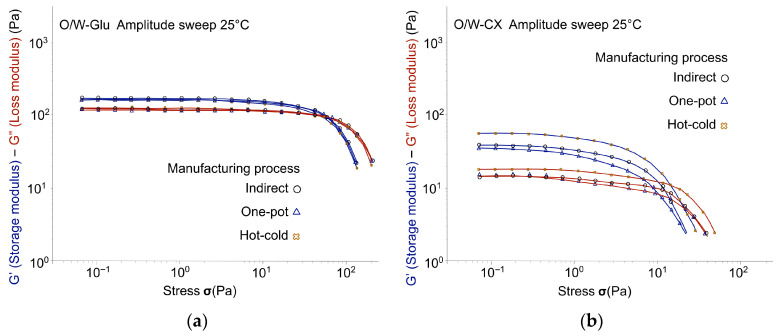
Oscillatory stress sweep: G′ and G″ moduli across manufacturing processes (Day 7, at 25 °C ± 2 °C): (**a**) Emulsions with glucomannan: O/W-Glu; (**b**) Emulgels with cross-linked xanthan gum: O/W-CX.

**Figure 8:**
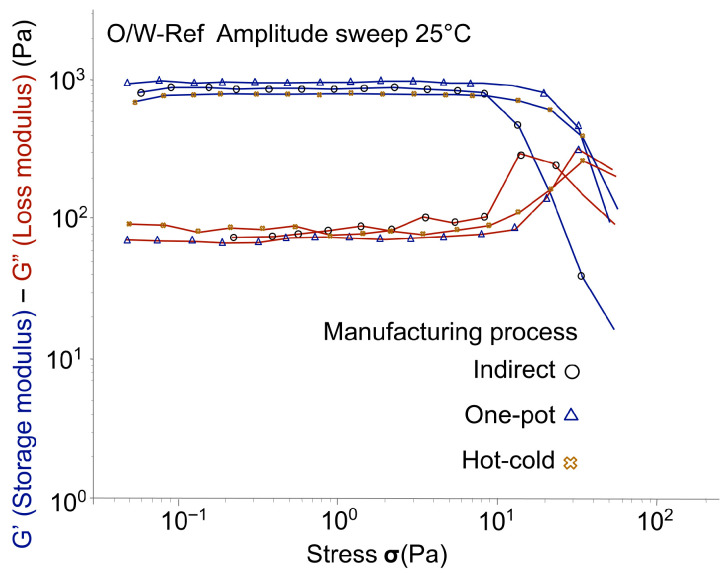
Oscillatory stress sweep (Day 7, at 25 °C ± 2 °C): G′ and G″ moduli across manufacturing processes for the synthetic reference (O/W-Ref).

Figure 6, Figure 7 and Figure 8 tracked the G′ and G″ moduli resulting from the oscillatory stress sweep for each polymer, with the data for different manufacturing processes superimposed. The relative magnitudes of G′ and G″ provide an initial, overall view of the emulsion’s elastic character. The manufacturing process did not significantly change the overall emulsion’s viscoelastic profile, with the exception of emulgels stabilized by co-processed acacia gum/xanthan gum (O/W-AX). For O/W-AX, the curves obtained indicated a critical decrease in the linear viscoelastic domain for the one-pot process, as highlighted by arrows in Figure 6a. Despite the higher G′ and G″ values, this shift suggested a reduced resistance of the gel structure to oscillatory stress, which may compromise its stabilizing efficacy, particularly in applications requiring the suspension of solid particles such as mineral sun filters or pigments [29].

The combined analysis of G′ and tan δ parameters allowed for relative comparisons (Table 3). Viscoelastic properties of the emulsion depended on the selected polymer.

Across all manufacturing processes, emulsions containing glucomannan (O/W-Glu; Figure 7a) did not exhibit a distinctive elastic character. The resulting profile, a weakly structured fluid (tan δ ≌ 0.7; Table 3), was consistently close to the liquid-like limit. The results were consistent with glucomannan functioning as a thickener. The instability of the emulsion without polymer did not allow for the detection of potential synergistic effects between the emulsifier and the polymer. However, data on gels (more precisely on hydrogels and sols in water) provided information complementary to the literature, as variability in polymer sources could induce variations in the response (Table A3, Appendix A). Dispersion of the glucomannan at the same concentration in water resulted in a typical liquid-like, highly viscous colloidal solution (Tan δ ≌ 0.9), confirming its driving effect on the O/W-Glu emulsion profile as a flow modifier rather than a gelling agent, in contrast to the other biopolymers tested.

Emulgels with tara gum (O/W-Tara; Figure 6b) showed a weak gel structure as indicated by tan δ, ranging from 0.43 to 0.65, but with a noticeable G′ absolute value (213.61 Pa; Table 3). The hot-cold process led to the weakest emulgel structure, evidenced by a significant decrease in G′ (from 213.61 Pa for the indirect process to 120.22 Pa) and an increase in tan δ (from 0.43 to 0.65). Although data for the aqueous dispersion were not available at the corresponding concentration, a 1% dispersion yielded a typical viscous colloidal solution (Tan δ ≌ 1.2; Table A3, Appendix A). These results suggested that the combination with the emulsifier provided enhanced structure, consistent with findings reported in the literature [30].

Although not reaching the same level as the synthetic polymer reference (O/W-Ref; Figure 8), emulgels with co-processed acacia gum/xanthan gum (O/W-AX; Figure 6a) and cross-linked xanthan gum (O/W-CX; Figure 7b) imparted a dominant elastic character, related to gel structure in the continuous phase. These results are consistent with weak gels obtained when dispersed in water (Tan δ ≌ 0.3–0.4; Table A3, Appendix A). Despite significant variations in G′ and G″ values with the one-pot process, the structure of O/W-AX seemed stable across manufacturing processes when looking at close values of tan δ. However, as noted at the beginning of this section, a drastic reduction of the linear domain with the one-pot process suggested caution. For the experimental xanthan gum (O/W-CX; Figure 7b), the emulgel structure seemed relatively similar across the manufacturing processes; a slight decrease in tan δ was observed with the hot-cold process.

To further characterize the structure, oscillatory shear frequency sweeps were conducted on the O/W-AX, O/W-Tar, and O/W-Ref emulgels. The frequency sweep for the co-processed acacia-xanthan (O/W-AX) manufactured via the one-pot process was omitted due to a significant drop in LVR. For all three emulgels, the dominant elastic behavior (G′ > G″) was observed across the frequency range (0.1–10 Hz), with no crossover point, confirming a gel structure. Therefore, the frequency dependence of G′ was modeled using a power-law relationship, framing the system within the theoretical Weak Gel Model [31]:

G′(ω) = K′·ω^n′^ (1)

**Figure A1:**
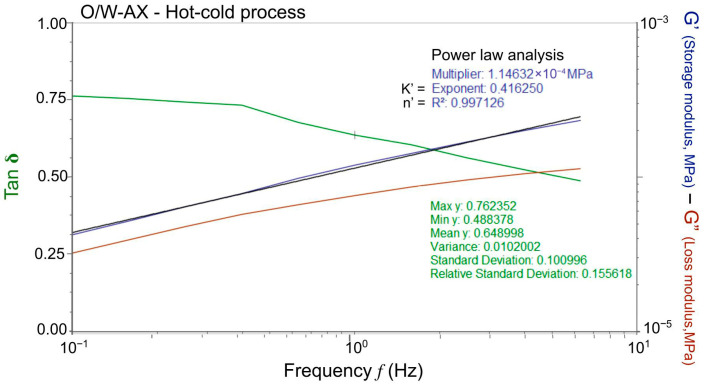
Stability of the elastic structure across frequency variations from 0.1 to 10 Hz. Emulgel with co-processed acacia gum/xanthan gum: O/W-AX manufactured with hot-cold procedure processes (Day 7, at 25 °C ± 2 °C).

**Figure A2:**
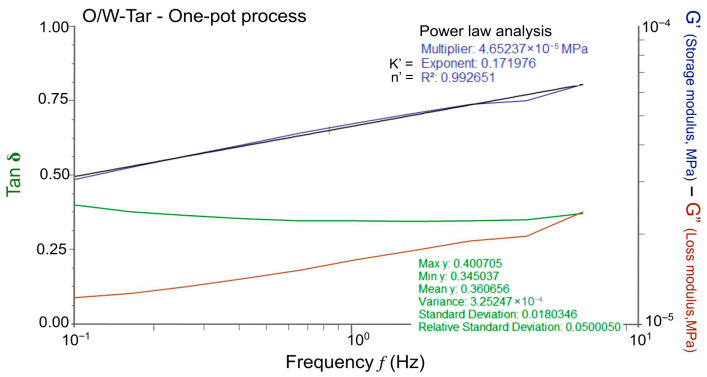
Stability of the elastic structure across frequency variations from 0.1 to 10 Hz. Emulgel with tara gum manufactured with a one-pot procedure (Day 7, at 25 °C ± 2 °C).

**Figure A3:**
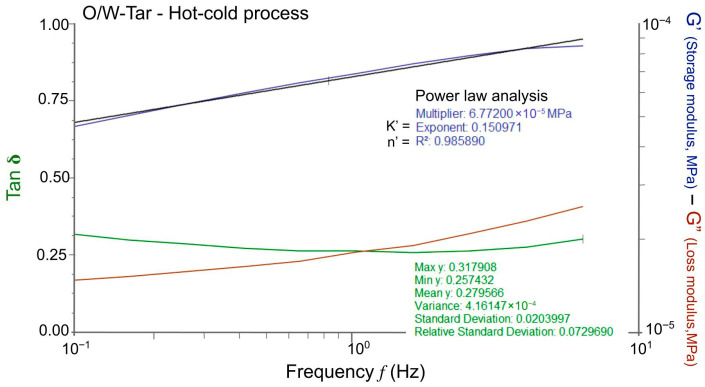
Stability of the elastic structure across frequency variations from 0.1 to 10 Hz. Emulgel with co-processed acacia gum/xanthan gum manufactured with hot-cold procedure processes (Day 7, at 25 °C ± 2 °C).

Following this approach, used to describe microstructured networks of foods, the equation parameters provide direct physical insight into the gel’s microstructure. The coefficient K′ reflects the network strength (referenced A in weak gel literature). It represents the number and strength of molecular interactions within the emulgel. The exponent n′ describes the degree of network connectivity and relaxation behavior, and is referred to as the coordination number z (n′ = 1/z) in the weak gel model). n′ value close to 0 indicates a highly organized solid-like network with frequency independence. A larger n′ value indicated a weak, more fluid-like network (see Table 4 and the curves for biopolymer emulgels in Appendix B: Figure A1, Figure A2 and Figure A3). As expected, the synthetic polymer emulgel exhibited a dense, highly organized three-dimensional solid-like network confirmed by a high K′ value, n′ value close to zero, which was similar across the three manufacturing processes. The indirect and hot-cold processes also resulted in O/W-AX emulgels with similar networks. For O/W-Tar emulgels, the K′ value was lower when prepared with the one-pot and hot-cold processes, consistent with the lower G′ value and increased tan δ previously observed at 1 Hz frequency.

Compared to the indirect process, the one-pot process resulted in approximately a 19% reduction in network strength while maintaining a constant connectivity exponent (n′). This indicated a uniform softening of the matrix: while the overall strength of the interactions was reduced, the fundamental 3D topology and coordination of the network were preserved. The hot-cold process resulted in a more substantial reduction in network strength (around 43%), accompanied by a concomitant increase in the connectivity exponent. Since G′ remained greater than G″ across the entire frequency range, this suggested that the gel was not disrupted. Rather than indicating formulation instability, this quantitative drop in K′ and rise in n′ suggested a ‘looseninG’ of the polymer matrix, leading to a less compact texture. From an applied perspective, this manufacturing process could be used to modulate the emulgel’s rigid structure into a softer, more manageable texture without compromising its macroscopic physical stability [32]. In practice, it could help to decrease the perception of a “jelly texture” during routine container handling (when the product is subjected to low-shear stress such as tilting) or when picking up the product. Sensory evaluations or a deeper analysis of the texture should be conducted to confirm these expectations.

**Figure 9:**
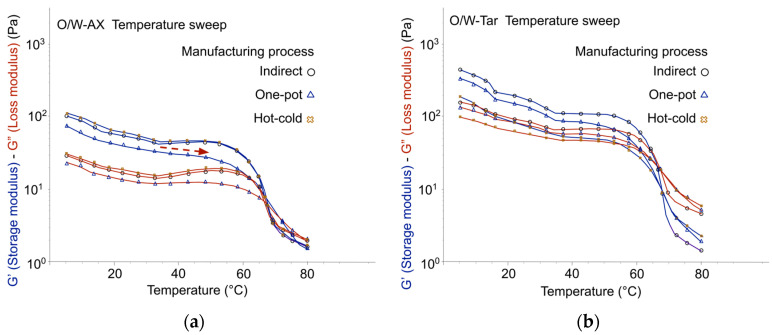
Stability of the elastic structure across temperature variations from 5 °C to 80 °C, Day 7: (**a**) Emulgels with co-processed acacia gum/xanthan gum: O/W-AX; (**b**) Emulgels with tara gum: O/W-Tar.

**Figure 10:**
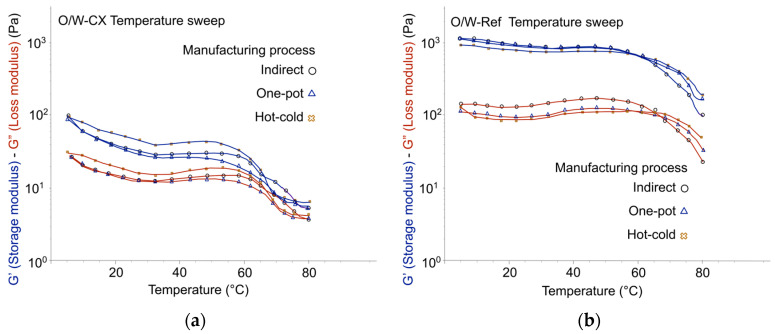
Stability of the elastic structure across temperature variations from 5 °C to 80 °C, Day 7: (**a**) Emulgels with cross-linked xanthan gum: O/W-CX; (**b**) Emulgels with the synthetic reference: O/W-Ref.

In addition, to assess emulgels structural stability, temperature ramp experiments (from 5 °C to 80 °C) were conducted with O/W-AX, O/W-Tar, O/W-CX, and O/W-Ref. All tested emulsions maintained structural integrity up to 55–60 °C (Figure 9 and Figure 10), a finding consistent with their macroscopic stability at one month at 45 °C. However, oscillatory experiments, which have established correlations for O/W emulsion stability [23,33], consistently suggested that the one-pot process should be avoided for the co-processed acacia/xanthan gum (O/W-AX). Figure 9a confirmed this by showing a reduced gelled structure stability, where the premature loss of storage modulus (G′) indicated a heightened potential for long-term instability. Conversely, the viscoelastic profiles for Tara gum, glucomannan, and cross-linked xanthan gum suggested similar structural integrity across the tested processes, and despite their generally lower level of emulsion structuring than the synthetic reference, one-pot or hot-cold processes showed no cause for concern regarding long-term stability.

### 2.4. Impact of Processing on Energy Consumption

**Figure 11:**
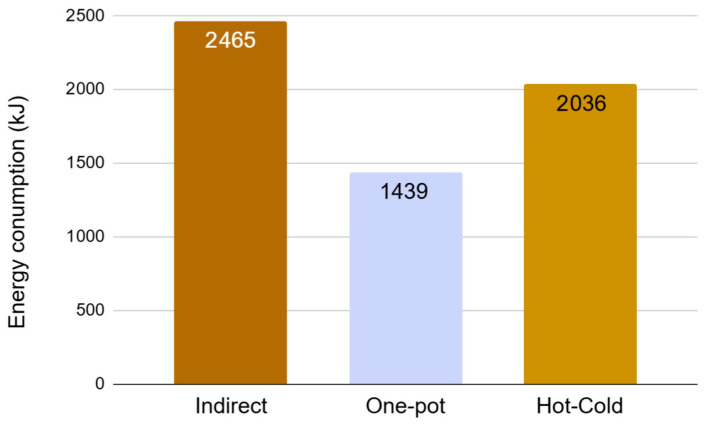
Total energy consumption for manufacturing 200 g of O/W-Ref emulgel with indirect, one-pot and hot-cold processes, expressed in kilojoules.

The successful application of these manufacturing methods to the tested biopolymers significantly broadens their scope of application. Therefore, indicative energy consumption measurements were conducted using the current emulgel with the synthetic reference (O/W-Ref) as a model to quantify the benefits. The data presented in Figure 11 were calculated by summing the consumption at each stage by the different electrical devices. The evaluation of the indirect process confirmed that the heating step is the main energy contributor, representing approximately 90% of the total energy consumed. The results showed a clear reduction in energy consumption for both the one-pot and hot-cold processes compared to the indirect process. Specifically, the one-pot process achieved a reduction of around 40%, and the hot-cold process a reduction of around 20%, compared to the indirect process. Although these laboratory-scale measurements were only indicative (performed once on a small quantity) and required confirmation in conditions closer to industrial production, the results suggested both processes are energy-efficient. The observed difference between these reduction levels and the higher thermal energy savings reported in the literature (e.g., 82% savings for the hot-cold process) can be explained by the high-melting-point emulsifier and the specific laboratory conditions involved in this study. While the benefits of the hot-cold process were previously shown with xanthan gum, the current work extends its applicability to other biopolymers. To our knowledge, the impact of one-pot processes on energy consumption has not been reported in the literature. However, its ability to provide physical stability and a similar sensory profile to the classical manufacturing process was previously demonstrated using a different emulsifying system [17]. The current work extends those investigations by comparing the rheological properties.

## 3. Conclusions

This work demonstrated the applicability of sustainable one-pot and hot-cold manufacturing processes to emulgels stabilized by biopolymers with diverse structural architectures (linear, branched chains, cross-linking, and complex blend structures). These energy-reducing and logistics-simplifying methods maintained the stability or bulk flow properties of most formulations after one month. The influence of the process, when observed, was polymer-specific and often beneficial. They successfully optimized flow properties for tara and cross-linked xanthan gums, though the one-pot method proved unsuitable for a co-processed Acacia/Xanthan blend at this dosage, requiring further investigation at lower concentrations. Ultimately, these findings broaden the scope of sustainable cosmetic manufacturing beyond traditional xanthan gum, offering promising pathways to reduce the industry’s environmental footprint. Future work should prioritize larger-scale trials, long-term stability validation, and in vivo sensory evaluations to confirm industrial viability and real-world performance.

## 4. Materials and Methods

### 4.1. Materials

The oil phase consisted of caprylic/capric triglyceride (CAS No. 9000-01-5, Dub MCT 5545, Stearinerie Dubois, Ciron, France). The glucolipid surfactant used was a cetearyl glucoside/cetearyl alcohol composition (CAS Nos. 661-19-8, 629-96-9, and 100231-68-3, respectively Montanov™ 68, Seppic, La Garenne Colombes, France). Deionized water was used for the aqueous phase. The polymers were sourced from Seppic (La Garenne Colombes, France): tara gum (INCI: *Caesalpinia spinosa* Gum, CAS No. 39300-88-4); co-processed acacia gum-xanthan gum (typical concentration 55%/45% *w*/*w*, where xanthan gum is coated by an acacia gum dispersion using spray drying technology; INCI: *Acacia senegal* Gum (and) Xanthan Gum, CAS Nos. 9000-01-5 and 11138-66-2, respectively); glucomannan extracted from the tubers of *Amorphophallus muelleri* (INCI: Glucomannan, CAS No. 11078-31-2); experimental xanthan gum cross-linked with sodium trimetaphosphate (STMP, CAS No. 7785-84-4); and the synthetic reference (INCI: Hydroxyethyl Acrylate/Sodium Acryloyldimethyl Taurate Copolymer). All polymers were supplied in powder form. The preservative system, which included the blends (INCI: phenoxyethanol and ethylhexylglycerin) and (INCI: phenylpropanol, caprylyl glycol, propanediol, and tocopherol), was purchased from Schulke & Mayr (Euxyl^®^ PE 9010 and Sensiva™ pa 40, Norderstedt, Germany). Triethanolamine (CAS No. 102-71-6) was obtained from Sigma-Aldrich (Saint-Louis, MO, USA).

### 4.2. Formulations Manufacturing Procedures

To assess the versatility of incorporating bio-based polymers, three distinct emulsion manufacturing processes were investigated. Direct addition of the polymer in powder form to the oil phase was selected to ensure a homogeneous hydration of the biopolymers, in the basic process (manufacturing process 1, called indirect). The emulsifying surfactant, supplied as solid pearls, required heating to 80 °C to be completely melted in the oil, consistent with the traditional manufacturing process. The control emulsion (without polymer) prepared with manufacturing process 1 was unstable seven days after manufacturing at room temperature. Emulsion manufacturing was performed at a 200 g laboratory scale using the same homogenizer (Silverson L4RT equipped with emulsor screen, Silverson, East Longmeadow, MA, USA). The pH was left at the initial value if it was between 5 and 7, or adjusted to be within this range if necessary.

#### 4.2.1. Indirect Process (Reference)

**Figure 12:**
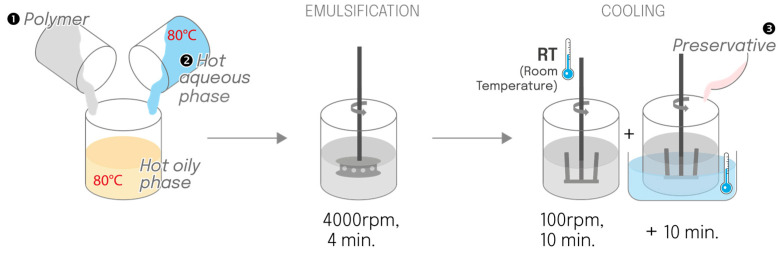
Schematic of the basic indirect manufacturing process, which served as a reference.

The aqueous and oil phases were heated separately to 80 °C. The polymer was added to the hot oily phase, followed by the addition of the hot aqueous phase (80 °C). Emulsification was performed using a rotor-stator turbine at 4000 rpm for 4 min. The emulsion cooled at room temperature under an anchor stirrer at 100 rpm for 10 min, followed by 10 min in a cold-water bath, and preservatives were added at around 30 °C (Figure 12).

#### 4.2.2. One-Pot Process

**Figure 13:**
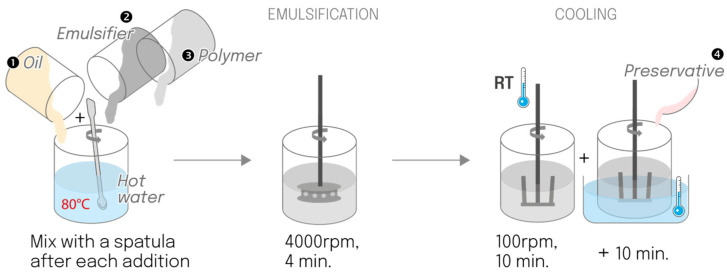
Schematic of a one-pot manufacturing process, for which all ingredients were added sequentially to the main tank (reduced cleaning operations and water consumption).

All ingredients (water, oil, surfactant, and polymer) were sequentially added to a single vessel and heated together to 80 °C, with mixing using a spatula after each addition. Emulsification was performed using a rotor-stator turbine at 4000 rpm for 4 min. The emulsion cooled at room temperature under an anchor stirrer at 100 rpm for 10 min, followed by 10 min in a cold-water bath, and preservatives were added at around 30 °C (Figure 13).

#### 4.2.3. Hot-Cold Process

**Figure 14:**
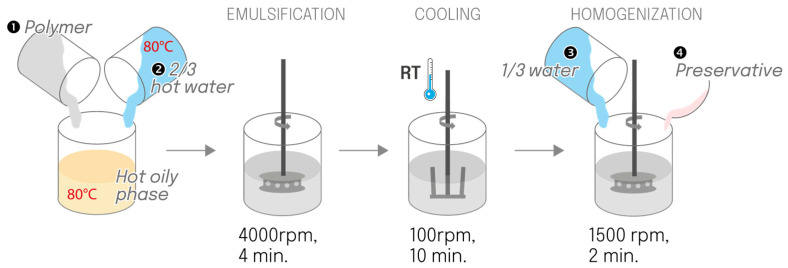
Schematic of the hot-cold manufacturing process, for which only two-thirds of the water was heated (reduced heating and cooling operations).

A concentrated “pre-emulsion” was prepared at 80 °C. The polymer was added to the hot oily phase (80 °C), followed by the addition of two-thirds of the hot water. Emulsification was performed using a rotor-stator turbine at 4000 rpm for 4 min. The emulsion cooled at room temperature under an anchor stirrer at 100 rpm for 10 min. Subsequently, the preservatives and the remaining water were added at room temperature to accelerate cooling, followed by homogenization using a rotor-stator turbine at 1500 rpm for 2 min (Figure 14).

### 4.3. Emulsion Stability and Characterization

The physical stability evaluation encompassed a one-month testing period under dual conditions in a closed environment away from light: room temperature (RT, 20 °C ± 2 °C) and elevated temperature (45 °C ± 5 °C). Samples at the elevated temperature were stored in a UF750plus lab oven (Memmert GmbH + Co.KG, Schwabach, Germany). Stability was evaluated at specific intervals—Day 7 and Month 1—on RT samples for visual appearance, pH, and Brookfield Viscosity. These parameters were measured once at each time point. The control at seven days after manufacturing was selected for complete stabilization of lamellar phase organization provided by the emulsifying system. Evaluations on 45 °C ± 5 °C samples included visual appearance at Day 7 and Month 1. Visual appearance included a qualitative assessment of flowability. If the formula flowed spontaneously upon turning the bottle over, it was described as pourable. The formulation was considered compact when it did not flow even after turning the bottle over and tapping the bottom. If the tapping triggered the flow, the formula was noted as pourable. The instrumentation included a SevenEasy pH meter (Metler-Toledo, Greifensee, Switzerland) and a Brookfield LV viscometer used to determine the apparent viscosity at defined speeds (spindle numbers 2 to 4, speed 6, Brookfield Engineering Laboratories Inc., Middleboro, MA, USA). This empirical, single-point measurement was used for quick quality-control comparisons at different times and to determine the macroscopic stability of the samples. A sample was deemed stable upon meeting the following criteria: the absence of macroscopic instability (including heterogeneous appearance, exudation, sedimentation, or phase separation), a maximum Brookfield Viscosity change of 20% relative to the Day 7 value, and a maximum pH change of ± 0.2 (thresholds related to the standard deviation of the measurement devices).

Microstructure was analyzed using an optical microscope (×40 magnification; microscope Eclipse Ni-U, Nikon Europe B.V., Amstelveen, The Netherlands) to evaluate droplet distribution and visualize lamellar phases around oil droplets. Analysis was performed on samples stored at room temperature on Day 7. Qualitative observations were conducted using both white and polarized light.

### 4.4. Rheological Analysis

Fundamental rheological characterization was performed using a Discovery Hybrid Rheometer (DHR2; TA Instruments, New Castle, DE, USA). This instrument served as both a stress-controlled and rate-controlled rheometer, allowing for rotational tests (to determine flow curves and yield stress) and oscillatory tests (to determine viscoelastic properties). Experiments were conducted seven days after manufacturing. All samples were allowed to equilibrate on the plate for 120 s prior to the start of measurements. Rheological characterization of the emulsions (rotational and oscillatory) used a Cone-Plate geometry (40 mm plate diameter, 2° angle cone, 52 µm truncation gap), following initial trials with the reference polymer. Although the central truncation gap approaches the particle size for emulsions with larger droplets (e.g., O/W-Glu hot-cold), the gap scales linearly with the radius, reaching 1393 µm at the periphery, which is significantly larger than the particle size (>10×). Since measured torque is dominated by stress at the outer radius (R^3^ scaling), potential confinement effects (jamming or bridging) at the center were considered negligible. This conclusion was supported by reproducibility between duplicates, smooth curves, and by the viscoelastic data, which showed liquid-like behavior for O/W-Glu across all processes, effectively ruling out the solid-like artifacts typically induced by particle confinement. To avoid any doubt, measurements on aqueous gels were performed using a serrated 40 mm plate/plate with a 1000 µm gap.

Flow experiments were conducted at 25 °C ± 2 °C to determine the yield stress and flow behavior of the formulations. Yield stress, defined as the minimum stress required to initiate flow (measured in Pascal, Pa), was quantified by applying a shear stress ramp from 0 to 200 Pa over 60 s using a steady-state protocol (onset point analysis: determination by tangent intersection using a log Viscosity/log Shear stress scale) [34]. The flow profile was characterized by applying a linear shear rate ramp from 0 to 1200 s^−1^ over 120 s (0 to 1000 s^−1^ for the aqueous gels), maintaining the maximum shear rate for 60 s, and then decreasing the shear rate back to 0 over 120 s. The measurements were performed in duplicate; a third replicate was implemented if variability in the results was observed. For simplified analysis, the results were presented as viscosity variations as a function of shear rate. Shear-thinning behavior was quantified using the Herschel–Bulkley model for formulations with a yield stress and the Williamson model for those without.

Herschel–Bulkley model:

(2) $$\tau =\tau_{0}+K{\dot{\gamma }}^{n}$$

where *τ* represents shear stress (Pa), *τ*_0_ represents the yield stress (Pa), *K* represents the consistency index (Pa·s^n^), $\dot{\gamma }$ represents the shear rate (s^−1^), and n represents the flow behavior index (dimensionless).

Williamson model:

(3) $$\eta =\frac{\eta_{0}}{1+{\left(k\dot{\gamma }\right)}^{n}}$$

where $\eta_{0}$ represents the zero-shear viscosity (Pa·s; the viscosity at the low shear rate newtonian plateau), $\dot{\gamma }$ represents the shear rate (s^−1^), k represents a time constant (s) determining the shear rate at which the transition from Newtonian to shear-thinning behavior occurs, and n represents the flow behavior index (dimensionless).

Both models yield a dimensionless Flow Behavior Index (n, also called rate index) that characterizes the slope of the shear-thinning region: a value closer to zero indicates more pronounced shear-thinning behavior, while an index closer to 1 suggests less pronounced shear-thinning behavior, approaching that of a Newtonian fluid (*n* = 1) [35]. The area enclosed by the upward and downward flow curves (the hysteresis loop area) served to compare thixotropic behavior [36] between the different processes. Viscosity was reported at a shear rate of 16.3 s^−1^. This specific value was chosen for two reasons: it corresponds to the typical shear conditions encountered during product pouring from packaging (10s^−1^–50 s^−1^), and it ensured a direct and consistent comparison across all samples during the initial use step.

Oscillatory rheology experiments were conducted to characterize the viscoelastic properties of the formulations and to assess the influence of the manufacturing process on their microstructure. Measurements were performed in duplicate; a third replicate was implemented if variability was observed. Initially, the structural properties were evaluated at 25 °C ± 2 °C using an amplitude sweep (0–100 Pa) at a fixed oscillation frequency of 1 Hz to determine the linear region. For formulations exhibiting significant viscoelastic behavior, a frequency sweep was subsequently applied from 0.1 to 10 Hz at a fixed stress of 0.6 Pa. Thermal stability of the emulgel structure was determined using temperature ramp experiments (5 °C to 80 °C) under fixed oscillatory conditions (1 Hz, 1 Pa). The storage modulus (G′, representing elastic behavior) and tan δ were monitored as indicators of the formulation’s structure. These parameters were calculated from the average values within the linear viscoelastic region (LVR). A lower tan δ value indicates a more structured product.

Data analysis was performed using TRIOS™ software (version 5.1.1.46572) associated with the DHR2 rheometer. Data were presented as Mean ± Standard Deviation (SD).

### 4.5. Energy Consumption Measurements

Energy consumed for the laboratory-scale manufacturing of emulgel with the synthetic reference (O/W-Ref) was measured for the three processes: indirect, one-pot, and hot-cold, using the same standardized equipment (One replicate). The energy was measured using a Wattmeter (Voltcraft Energy Check 300, Conrad Electronic, Hirschau, Germany). The Wattmeter was connected to the electrical outlet of each device. The effective consumption time was monitored with a stopwatch, allowing the calculation of energy in kilojoules (energy = power in Watt × time). Total energy consumption was subsequently calculated by summing the consumption at each stage (heating, emulsification, and cooling) by the different electrical devices. The heating step was performed using a water bath (Julabo Pura 10, capacity 10 L, Julabo GmbH, Seelbach, Germany). To more closely simulate industrial manufacturing conditions for the indirect and hot-cold processes, the measurement accounted for the energy required to heat each emulsion phase separately, unlike classic laboratory procedures, where both phases are typically heated simultaneously in a water bath.

## Figures and Tables

**Table 1:** Emulsion Characteristics and Stability Summary: pH and Brookfield viscosity were performed at 20 °C ± 2 °C. Visual appearance was assessed at 20 °C ± 2 °C and 45 °C ± 5 °C.

Ingredients (%*w*/*w*)		O/W-AX	O/W-Tar	O/W-Glu	O/W-CX	O/W-Ref
Water		Up to 100	Up to 100	Up to 100	Up to 100	Up to 100
Caprylic/capric triglyceride		10.0	10.0	10.0	10.0	10.0
Glucolipid emulsifier		3.0	3.0	3.0	3.0	3.0
*Acacia senegal* Gum & Xanthan Gum		1.2	-	-	-	-
*Caesalpinia spinosa* Gum		-	0.8	-	-	-
Glucomannan		-	-	1.0	-	-
Cross-linked xanthan gum		-	-	-	0.5	-
Synthetic reference		-	-	-	-	0.5
Preservative *		1	1	1	1	1
Triethanolamine (50% solution)		-	-	-	as required	-
Characteristics 7 Days after manufacturing at room temperature						
Visual appearance **		liquid white	pourable white	compact white	liquid white	compact white
pH	Indirect	7.0	6.8	5.5	6.2	6.6
	One-pot	7.0	6.7	5.4	6.2	6.4
	Hot-cold	7.0	6.6	5.6	6.2	6.6
Brookfield viscosity (Pa·s)	Indirect	6.50	17.30	61.20	9.90	34.35
	One-pot	5.90	19.00	51.80	9.30	38.50
	Hot-cold	6.86	14.76	48.00	11.10	30.59
Characteristics 1 Month after manufacturing at room temperature						
Visual appearance **		liquid white	pourable white	compact white	liquid white	compact white
pH	Indirect	6.9	6.6	5.4	6.0	6.7
	One-pot	6.9	6.6	5.3	6.0	6.3
	Hot-cold	6.8	6.6	5.4	6.0	6.4
Brookfield viscosity (Pa·s)	Indirect	7.46	17.20	53.50	11.30	34.35
	One-pot	6.30	24.00 ***	51.00	10.50	34.50
	Hot-cold	8.00	13.80	54.00	12.70	27.50
Stability―Month 1 at room temperature and 45 °C		Stable	Brookfield Viscosity increase for one-pot/Stable appearance and pH	Stable	Stable	Stable

* 0.5% of phenoxyethanol and ethylhexylglycerin blend combined with 0.2% of phenylpropanol and caprylyl glycol and propanediol and tocopherol blend; ** Visual appearance was assessed at room temperature and 45 °C: no instability was observed. *** The observed difference post-manufacturing significantly exceeds the inherent device error of approximately 20% when compared to Day 7 measurements.

**Table 2:** Impact of manufacturing process on flow curve characteristics of emulsions depending on the polymer nature from Herschel–Bulkley (Equation (2)) when a yield stress was identified and from the Williamson model (Equation (3)) when no yield stress could be measured. Measurements were performed at 25 °C ± 2 °C on Day 7 after manufacturing.

Formula	Flow Curve Analysis	Indirect Process	One-Pot Process	Hot-Cold Process
O/W-AX	Yield stress (Pa)	2.77 ± 0.11	2.84 ± 0.15 ≌ 3	2.75 ± 0.12
	Flow behavior index	0.56 ± 0.01	0.62 ± 0.02	0.57 ± 0.02
	Viscosity at 16.3 s^−1^ (Pa·s)	0.70 ± 0.01	0.50 ± 0.11 *	0.75 ± 0.03 *
	Hysteresis loop area (Pa/s)	4919.73 ± 147.57	3931.74 ± 117.95 *	5170.95 ± 103.4
O/W-Tar	Yield stress (Pa)	3.61 ± 0.38	3.01 ± 0.12	3.20 ± 0.12
	Flow behavior index	0.05 ± 0.00	0.06 ± 0.00	0.12 ± 0.01
	Viscosity at 16.3 s^−1^ (Pa·s)	3.69 ± 0.13	3.50 ± 0.01	3.55 ± 0.07
	Hysteresis loop area (Pa/s)	15,987.00 ± 799.51	16,019.10 ± 640.76	11,272.30 ± 563.6 *
O/W-Glu	Yield stress (Pa)	None	None	None
	Flow behavior index	0.81 ± 0.00	0.812 ± 0.00	0.80 ± 0.00
	Viscosity at 16.3 s^−1^ (Pa·s)	8.52 ± 0.11	8.18 ± 0.26	7.89 ± 0.11
	Hysteresis loop area (Pa/s)	Not significant	Not significant	Not significant
O/W-CX	Yield stress (Pa)	23.03 ± 0.08	11.78 ± 0.61 *	17.14 ± 1.23 *
	Flow behavior index	0.68 ± 0.00	0.45 ± 0.00 *	0.47 ± 0.01 *
	Viscosity at 16.3 s^−1^ (Pa·s)	1.52 ± 0.01	1.51 ± 0.00	1.81 ± 0.06 *
	Hysteresis loop area (Pa/s)	2797.76 ± 828.52	2048.71 ± 247.59 *	3790.11 ± 642.69 *
O/W-Ref	Yield stress (Pa)	11.07 ± 0.25	17.59 ± 0.86	14 ± 0.65
	Flow behavior index	0.40 ± 0.02	0.43 ± 0.02	0.41 ± 0.02
	Viscosity at 16.3 s^−1^ (Pa·s)	5.69 ± 0.31	4.70 ± 0.07 *	4.54 ± 0.38 *
	Hysteresis loop area (Pa/s)	30,072.70 ± 75.66	25,847.80 ± 75.66 *	19,016.80 * ± 75.66

* Values considered significantly different from those obtained with the indirect process.

**Table 3:** Impact of manufacturing process on viscoelastic moduli and tan δ depending on the polymer nature. Measurements were performed at 25 °C ± 2 °C on Day 7 after manufacturing.

Formula	Viscoelasticity Analysis	Indirect Process	One-Pot Process	Hot-Cold Process
O/W-AX	G′ (Pa)	55.79 ± 4.50	116.97 ± 57.95 *	62 ± 5.87
	G″ (Pa)	15.25 ± 0.85	37.75 ± 15.13 *	16.87 ± 0.55
	tan δ	0.28 ± 0.02	0.38 ± 0.07	0.29 ± 0.05
O/W-Tar	G′ (Pa)	213.61 ± 3.59	154.02 ± 8.36 *	120.22 ± 7.18 *
	G″ (Pa)	92.55 ± 0.88	78.92 ± 1.36 *	77.72 ± 1.01 *
	tan δ	0.43 ± 0.01	0.51 * ± 0.02	0.65 ± 0.03 *
O/W-Glu	G′ (Pa)	161.96 ± 12.26	155.03 ± 9.13	164.14 ± 6.83
	G″ (Pa)	123.19 ± 5.59	113.76 ± 4.06	121.28 ± 3.52
	tan δ	0.75 ± 0.05	0.74 ± 0.03	0.76 ± 0.05
O/W-CX	G′ (Pa)	34.95 ± 3.75	30.78 ± 4.16	52.56 ± 5.06 *
	G″ (Pa)	14.12 ± 0.90	14.43 ± 1.26	18.05 ± 0.76 *
	tan δ	0.40 ± 0.03	0.43 ± 0.02	0.35 ± 0.02 *
O/W-Ref	G′ (Pa)	998.83 ± 28.05	992.52 ± 43.40	770.08 ± 32.30 *
	G″ (Pa)	100.03 ± 10.67	76.74 ± 5.54 *	84.49 ± 12.46 *
	tan δ	0.11 ± 0.03	0.08 ± 0.02	0.14 ± 0.09

* Values considered significantly different from those obtained with the indirect process.

**Table 4:** Viscoelastic properties of emulgels/emulsions under frequency sweep. Measurements were performed at 25 °C ± 2 °C on Day 7 after manufacturing.

Emulgel *	Viscoelasticity Analysis	Indirect Process	One-Pot Process	Hot-Cold Process
O/W-AX	K′	63.16 ± 0.36	Not done	68.32 ± 0.42
	n′	0.16 ± 0.00	Not done	0.16 ± 0.01
O/W-Tar	K′	200.33 ± 18.00	161.79 ± 3.19	114.63 ± 9.17
	n′	0.27 ± 0.00	0.30 ± 0.00	0.41 ± 0.02
O/W-Ref	K′	897.11 ± 96.09	965.24 ± 28.96	824.86 ± 41.24
	n′	0.05 ± 0.00	0.05 ± 0.00	0.05 ± 0.00

* Completion of this analysis for O/W CX requires a new batch to be prepared for testing.

## Data Availability

The data that support the findings of this study are available from the corresponding author upon request. The data are not publicly available due to privacy restrictions.

## Abbreviations

The following abbreviations are used in this manuscript:

INCI	International Nomenclature of Cosmetic Ingredients
LVR	Linear Viscoelastic Region
O/W	Oil-in-Water
O/W-AX	Emulgel stabilized with co-processed acacia gum/xanthan gum blend
OW-Tar	Emulgel stabilized with tara gum
OW-Glu	Emulsion stabilized by glucomannan
OW-CX	Emulgel stabilized by cross-linked xanthan gum
OW-Ref	Emulgel stabilized by synthetic polymer reference

## Appendix A

The following tables summarize data on simple dispersions of polymers in water: gels/sols (Polymer X% + same combination of preservatives as in the O/W emulsions 1% + water up to 100%). Dispersions were realized under mechanical agitation (Agitation Turbotest 33/300 equipped with a serrated disc, VMI-Rayneri, Epe, The Netherlands) until a smooth gel appearance was obtained.

### Appendix A.1. Determination of Time Required to Obtain a Smooth Dispersion Appearance

The powder was sprinkled gradually under agitation. Agitation was initially set to 400 rpm to create a vortex, then increased to 800 rpm as viscosity increased. Agitation was maintained until no particulates (lumps) were detected. Homogeneity was determined through visual inspection for the absence of visible aggregates or lumps. Bulk consistency was verified by spatula extraction.

**Table A1:** Time required to obtain a smooth dispersion at room temperature (20 °C ± 2 °C), expressed in minutes.

Polymer	AX at 1%	Acacia Gum * at 1%	Xanthan Gum at 1%	Tar at 1%	Glu at 1%
Dispersion time (range of 5 replicates in min)	3 to 5	3 to 5	30 to 40	15 to 30	15 to 30

* Acacia gum dispersion was liquid (Brookfield viscosity < 0.050 Pa·s).

### Appendix A.2. Rheology Data on Aqueous Dispersions

The 0.8% tara dispersion could not be measured due to a precipitate formed on Day 7. Similarly, Ref at 0.5% was not fully stable. The results from the closest stable concentration were reported. Table A2 summarizes indicators collected in flow, using the Herschel-Bulkley model (Equation (2)) for yield stress fluids and the Williamson model (Equation (3)) when no yield stress could be detected.

**Table A2:** Flow curve characteristics of aqueous “gels” per polymer type, at 25 °C ± 2 °C on Day 7 after manufacturing.

Flow Curve Analysis	AX at 1%	Tar at 1%	Glu at 1%	CX at 0.5%	Ref at 0.8%
Yield stress (Pa)	2.68 ± 0.03	Not significant	No	1.40 ± 0.00	29.47 ± 1.81
Flow behavior index	0.34 ± 0.02	0.79 ± 0.01	0.81 ± 0.00	0.36 ± 0.03	0.33 ± 0.01
Thixotropy	Not significant	Not significant	Not significant	Not significant	Not significant
Brookfield Viscosity (Pa·s)	7	6.8	5.5	6.2	6.6

**Table A3:** Viscoelastic properties of aqueous polymer “gels” per polymer type, at 25 °C ± 2 °C on Day 7 after manufacturing.

Viscoelasticity Analysis	AX at 1%	Tar at 1%	Glu at 1%	CX at 0.5%	Ref at 0.8%
G′ (Pa)	≌4.93 ± 0.08	16.1 ± 0.16	48.5 ± 4.5	20.8 ± 0.31	772.79 ± 23.80
G″ (Pa)	1.55 ± 0.75	19 ± 0.10	44.8 ± 2.3	8.48 ± 0.28	96.93 ± 8.59
tan δ	0.31 ± 0.02	≌1.2 1.18 ± 0.02	0.92 ± 0.01	0.41 ± 0.02	0.12 ± 0.01

## Appendix B

Figure A1, Figure A2 and Figure A3 report the curves obtained from frequency sweeps for the emulgels stabilized by biopolymers. Frequency sweeps were performed within LVR over a range of 0.1–10 Hz at a constant strain (5%). The curves illustrate the analysis of G′ as a function of frequency by a power law model, which is explained in Section 2.3, and show the calculated indicators reported in Table 4.
